# Investigating the Status of Biological Stimuli as Objects of Attention in Multiple Object Tracking

**DOI:** 10.1371/journal.pone.0016232

**Published:** 2011-03-31

**Authors:** Lee H. de-Wit, Carmen E. Lefevre, Robert W. Kentridge, Geraint Rees, Ayse P. Saygin

**Affiliations:** 1 University of Leuven, Leuven, Belgium; 2 Durham University, Durham, United Kingdom; 3 University College London, London, United Kingdom; 4 University of California San Diego, La Jolla, California, United States of America; Max-Planck Institute of Neurobiology, Germany

## Abstract

**Background:**

Humans are able to track multiple simultaneously moving objects. A number of factors have been identified that can influence the ease with which objects can be attended and tracked. Here, we explored the possibility that object tracking abilities may be specialized for tracking biological targets such as people.

**Methodology/Principal Findings:**

We used the Multiple Object Tracking (MOT) paradigm to explore whether the high-level biological status of the targets affects the efficiency of attentional selection and tracking. In Experiment 1, we assessed the tracking of point-light biological motion figures. As controls, we used either the same stimuli or point-light letters, presented in upright, inverted or scrambled configurations. While scrambling significantly affected performance for both letters and point-light figures, there was an effect of inversion restricted to biological motion, inverted figures being harder to track. In Experiment 2, we found that tracking performance was equivalent for natural point-light walkers and ‘moon-walkers’, whose implied direction was incongruent with their actual direction of motion. In Experiment 3, we found higher tracking accuracy for inverted faces compared with upright faces. Thus, there was a double dissociation between inversion effects for biological motion and faces, with no inversion effect for our non-biological stimuli (letters, houses).

**Conclusions/Significance:**

MOT is sensitive to some, but not all naturalistic aspects of biological stimuli. There does not appear to be a highly specialized role for tracking people. However, MOT appears constrained by principles of object segmentation and grouping, where effectively grouped, coherent objects, but not necessarily biological objects, are tracked most successfully.

## Introduction

Each time we open our eyes, we are confronted with far more visual information than can be processed at once. The visual system therefore employs biases at multiple stages of information processing to ensure that critical stimuli receive more attention [1]. This process does not simply select spatial locations, since the allocation of attention to a spatial location can automatically lead to the attentional selection of objects at that location [2], [3]. More generally, it is well accepted that some form of ‘objecthood’ influences the allocation of attention and selection of targets [4].

There is however a degree of controversy regarding what exactly an object of attention is [5]. It is therefore highly pertinent to ask what features lead to the construction of an effective object for the purpose of attentional selection? Previous work has explored the role of simple contrast edges [6], the manner in which edges group into surfaces [7], Gestalt grouping principles [8], [9] and amodal completion [10], [11]. From a neural perspective, brain areas important in general grouping and completion phenomena, such as the Lateral Occipital Complex (LOC) [12], and inferior intraparietal sulcus (IPS) [13], have been argued to play a key role in the formation of the objects of attention [14]–[16]. Viewed collectively, these data point to a role of neural substrates involved in the computation of mid-level grouping factors or Gestalt principles in the formation of the objects selected by attention.

It is possible however, that factors beyond perceptual grouping play a role in defining the objects selected by attention. Here, we focused on the potential role of an important but previously understudied property in object-based attention: “biologicalness”. Thus, just as Scholl and colleagues studied the extent to which principles such as “connectedness” influence the ability of the human visual system to group stimuli as an object of attention tracking [9], so this paper seeks to understand whether stimuli that comprise biological objects are more effectively processed as units of attentional tracking.

The potential importance of this stimulus dimension was suggested not only by the special status that biological targets appear to have from very early in life [17], but also because other forms of attentional orienting are tuned to the biological importance of the stimuli. Directed gaze (but not geometric control stimuli) for example, automatically attracts attention [18]. Indeed a sensitivity to biological stimuli can also be seen in the context of saccade control, where upright faces have been reported to have a much more disruptive influence on involuntary saccade programming compared with inverted faces [19]. If the biological status of a stimulus can influence computations underpinning attention and eye movements, might it also have effects on object-based attention paradigms?

In the present paper, we employed the Multiple Object Tracking (MOT) paradigm, where observers are asked to track a set of moving target objects among identical distracters [9], [20]. We explored whether “biologicalness” is a factor that influences MOT, or whether the constraints on MOT are largely based on object segmentation and grouping mechanisms [3].

Pylyshyn has argued that MOT is underpinned by *proto-objects* that are computed on the basis of pre-conceptual mechanisms encapsulated in the early visual system [21]. Within this framework, proto-objects can be used as a kind of scaffold on which to frame conceptual knowledge, but this knowledge cannot be used in the formation of these objects. Accordingly, one would predict that higher-level properties of an object, such as its biological status could not play a role in MOT.

However, Pylyshyn's theory of MOT is not universally accepted. Some have pointed out that MOT may simply reflect a splitting of general attentional resources [22]. Indeed, recent research showed that individual differences in the initial selection and sustained tracking of multiple objects can be predicted by EEG components associated with more general attentional and working memory tasks, and highlighted the visual system's ability to individuate one object from another plays a central role in determining MOT performance [23]. Whether the biological status of an object can influence the ease with which it can be individuated as a target to be tracked remains an open question.

In the experiments presented here, we chose to operationalize “biologicalness” by employing point-light biological motion displays. Experiment 3 additionally also explores images of faces. Point-light biological motion figures consist of markers attached to the limbs of a person, about a dozen of which are sufficient to evoke a clear and vivid percept of a human body in motion [24]. Despite their simplicity and sparseness, the human brain is able to reconstruct these displays as biological objects in a network that includes temporal and frontal cortical areas [25]. Sensitivity to biological motion has been argued to be present from birth [17], and the mechanisms involved in processing biological motion may be distinct from those involved in other kinds of coherent motion, as well as non-biological object motion [26]–[30]. Compared to other possible body movement stimuli (e.g., video) point-light biological motion stimuli have been better studied, with abundant prior psychophysical data (see [31] for review) and are better suited for experimental manipulation. Point-light stimuli allow techniques such as inversion and scrambling to be used more straightforwardly to generate control stimuli that maintain local motion information, but change the percept considerably (see Methods).

The particular combination of MOT and biological motion allowed us to explore not only whether biological targets have a special status, but also whether MOT in particular was in some way adapted for ‘multiple people tracking’. The study of MOT is often motivated by the ecological validity of the task, highlighting the challenges of tracking animate entities moving in complex, crowded scenes along with distracters (e.g., “Imagine a primitive hunting party on the savannah stalking four weak gazelle amongst a larger herd” [32]). However, although a few studies had used animal images as targets [33]–[35], MOT for biological and non-biological targets had not been directly compared.

### Introduction: Experiment 1

We used point-light biological motion [24], plus well-established procedures for creating control stimuli that influence the perception of biological motion while maintaining local motion properties, namely inversion of presentation, and scrambling of the individual point-lights [25], [29], [36]–[38].

A pilot experiment with 12 subjects, provided evidence for a sensitivity to the inversion of biological motion stimuli in MOT. The data were collected with the same methods described below, employing only the upright and inverted biological motion conditions and all subjects gave written informed consent as below,. Tracking accuracy was significantly higher for upright compared with inverted point-light animations (70% in the upright and 66.6% in the inverted conditions (t(11) = 2.56, p = 0.026). This initial result suggested that biological information could play a role in MOT.

Experiment 1 employed 2.5 second periods of MOT to replicate this inversion effect with a larger sample, and compare it to the tracking of another point-light but non-biological target (the letter R). Since biological motion animations not only have the dynamics of natural body movements, but also coherent, familiar and recognizable form, we also manipulated the form of non-biological control stimuli (the letter ‘R’, composed of point-lights). If MOT is specialized for biological stimuli, a selective advantage for point-light biological stimuli may be found. The biological motion figures and letters however not only contain many structural differences, but are also very different in their internal motion profile. In order to gain some insight into the role of motion in determining differences between tracking letters and biological figures, a scrambled condition was also included. We therefore examined MOT using point-light biological motion stimuli and point-light letters, presented in upright, inverted and scrambled conditions.

#### Materials and Methods: Experiment 1

Thirty adults (18 females, mean age = 21, SD = 6.4) participated. The Durham University Ethics Committee approved the experimental protocol and informed written consent was obtained from the subjects after the nature and possible consequences of the study were explained to them. All participants had normal or corrected-to-normal vision.

**Figure 1:**
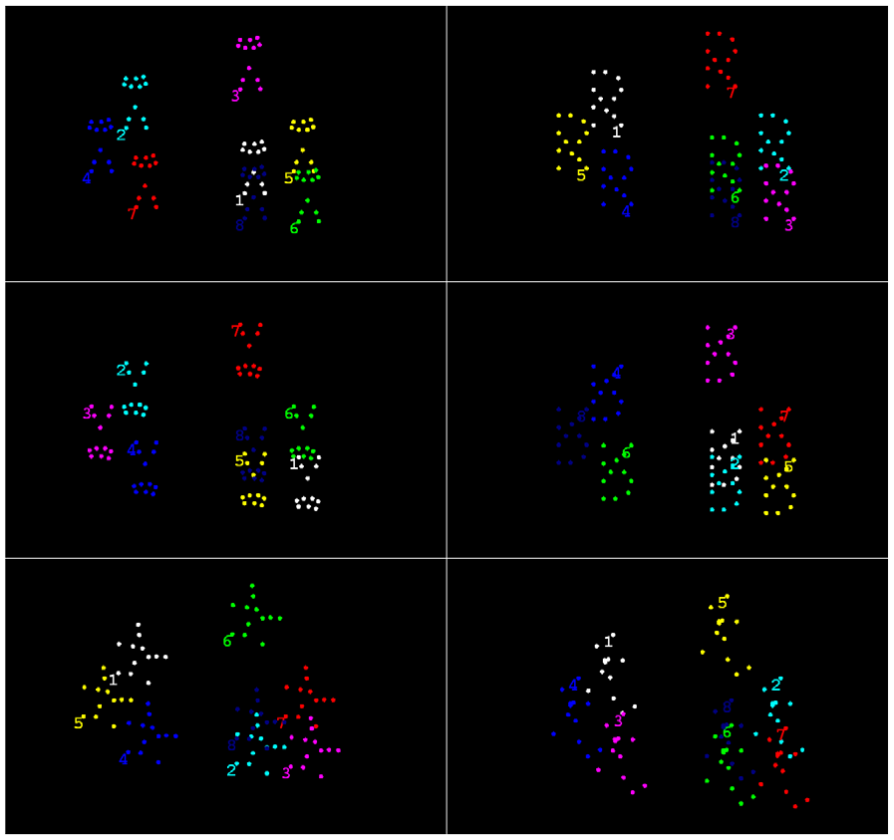
Frames from the biological (left panel) and non-biological (right panel) point-light stimuli in the upright, inverted and scrambled conditions. Participants were presented these displays at the end of each trial and were prompted to report the numbers corresponding to the four targets they had been tracking (see Methods and Materials: Experiment 1). During the trial, when the targets and distracters were moving around the screen, all points appeared in the same colour (white), apart from the first 30 frames in which the four targets flashed in red.

Stimuli were presented using Matlab (Mathworks, Natick, MA, USA) and the Psychophysics Toolbox [39], [40] on a 17 inch monitor, with 1280 by 800 resolution at 60 Hz. On each trial, eight individual objects were presented simultaneously on the screen. These comprised either biological or non-biological (letter) point-light stimuli. On each trial all eight stimuli were presented either upright, inverted or scrambled (Fig. 1).

Each individual biological motion stimulus comprised an animation that was created by videotaping an actor and then encoding the joint positions in the digitized videos [41]. We selected one specific animation depicting a star jump (or jumping jacks) as this action does not have an obvious implied direction of left/right motion (see Experiment 2 for an explicit manipulation of direction of motion). The joints were represented by twelve small white dots each subtending approximately 0.015 degrees of visual angle (participants viewed the screen at approximately 57 cm) against a black background. The height of each figure subtended 2 degrees of visual angle, the width varied (with the motion of the arm and leg joints) from 0.9–1.5 degrees. Each star jump consisted of 20 frames, which looped continuously throughout the trial.

Non-biological control stimuli were constructed using 12 points of light to form an uppercase letter R that subtended of 2 by 1.2 degrees of visual angle. The internal structure of the non-biological stimulus, unlike the star jump, did not change throughout the trial.

On each trial, participants were presented with eight point-light stimuli simultaneously. Each moved around a 10 by 8 degree area of the screen, following independent overlapping random paths. These paths were constructed off-line prior to the experiment using linked Bezier curves. The rate of change in these paths was constrained to avoid any sharp changes in direction. Each stimulus moved through 80 points defined along a curve. These points were constrained so that they were not separated by more than 0.21 degrees (8 pixels). A trial included 80 frames, and each frame was presented for 50 ms. For the first 30 frames, four of the stimuli flashed in red on alternate frames to indicate that they should be tracked as the targets, while the other four should be ignored. All targets and distracters then appeared white as they moved at an average speed of 4.15 degrees per second for 50 frames. At the end of a trial, each individual object changed to a different color, and a number was presented next to each stimulus in the same color (Fig. 1). Participants were required to report the numbers associated with the 4 stimuli they had been tracking by typing them on the keypad. Participants always had to enter 4 responses, and were instructed to guess if they were unsure. The tracking duration was 2.5 seconds, which is relatively short compared to most MOT studies.

The inverted stimuli were generated by vertically rotating each stimulus around its centre. Naïve observers typically cannot spontaneously recognize the inverted biological motion figures [38]. Scrambled biological motion animations were constructed by randomizing the starting positions of the points while keeping the motion trajectories of each individual dot intact. The starting positions were chosen randomly within a region such that the total area encompassed by each figure was similar to that of the upright figures. The scrambled animations therefore contained the same local motion cues but did not have the same global form as the upright biological motion animation and are instead perceived as somewhat coherently swirling set of dots. The non-biological (letter) stimuli were transformed into inverted and scrambled versions in the same manner as the biological motion figures.

Participants were presented with one block of biological and one block of non-biological targets, the order of which was counterbalanced across participants. Each block contained 60 trials with an equal number of upright, inverted and scrambled figures, presented in a random order.

#### Results: Experiment 1

**Figure 2:**
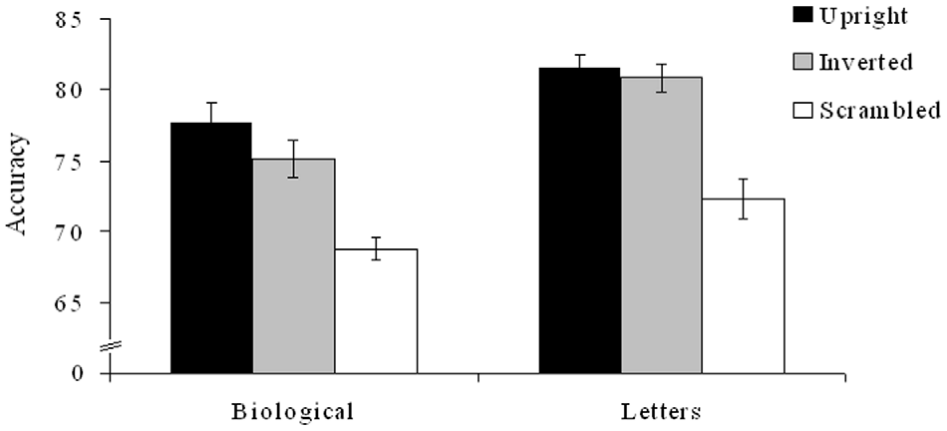
Accuracy (percentage correct) for tracking the upright, inverted and scrambled presentation of biological and letter targets. Error bars depict standard error. Scrambled stimuli were tracked less successfully compared with upright stimuli. In addition, inverted biological motion was tracked less accurately compared with upright biological motion (see Results: Experiment 1).

The data were analyzed using a repeated measures ANOVA, with 2 factors, stimulus type (biological, non-biological) and presentation type (upright, inverted, scrambled). The results, in terms of percentage correct responses, are presented in Figure 2. There was a main effect of stimulus type, such that the non-biological letter targets were in fact easier to track (F(1,29) = 62.74, p<0.001). There was also a main effect of presentation type (F(2,58) = 85.84, p<0.001). Scrambling the stimuli (relative to upright stimuli) had a substantial effect on tracking with significantly lower accuracy for scrambled stimuli (F(1,29) = 140.7, p<0.001). Scrambling did not interact with target type (p = 0.916).

The effect of inversion (relative to upright targets) was smaller but significant (F(1,29) = 5.197, p = 0.03). The interaction between the upright and inverted conditions for the different target types was not significant (F(29) = 1.38, p = 0.25), although as seen in Figure 2, the main effect of inversion was driven by the biological motion condition. Paired samples t-test comparing upright and inverted targets in the biological motion condition revealed a significant difference (t(29) = 2.58, p = 0.031); whereas the same was not the case for letter stimuli (t(29) = 0.71, p = 0.49).

#### Discussion: Experiment 1

We did not find evidence for superior tracking of biological targets. In fact, participants in Experiment 1 were better at tracking point-light letters compared with point-light biological motion figures. However, this difference was present also for scrambled stimuli, indicating it may not be the biological status or meaning *per se* that led to these results. For example, the difference may be due to the internal motion that disrupts the common fate of dots making up the targets in the biological motion condition [42]. On the other hand, a role of internal motion was not observed by van Marle and Scholl [43]. Since the dots making up each object in our Experiment 1 were spread over a wider area than the parts of the objects used in the latter study, it is possible that common fate plays a greater role in MOT when the distance between the to-be-grouped parts is larger.

Whilst point-light biological motion is perceived as a meaningful object, inverted and scrambled versions are not, but instead appear more as a group of swirling dots. We suggest that the large effect of scrambling on MOT could be due to disruption of grouping cues, consistent with previous work [9]. The smaller but still significant inversion effect on biological motion could be due to process of matching the stimuli to its canonical orientation and/or the loss of gravity cues [38], [44] (see Discussion).

Whilst the biological status of the targets seemed to play little role in the overall differences in accuracy between letters and biological targets and on the effect of scrambling, there was an inversion effect only for biological motion, replicating the findings of our pilot study with a different set of subjects. Experiment 2 sought to investigate whether other naturalistic or ecologically valid features of biological motion could also influence MOT. Experiment 3 further pursued inversion effects in MOT by utilizing another biological stimulus that exhibits an inversion effect, but does not involve internal motion cues (images of faces).

### Introduction: Experiment 2

We compared tracking performance for point-light walkers that had a motion translation consistent with their internal motion pattern, with point-light “moon-walkers”, whose local motion pattern suggested they were walking in one direction, while they in fact moved globally in the opposite direction.

#### Materials and Methods: Experiment 2

Twenty participants (9 Males, Mean age = 25, SD = 5) with normal or corrected-to-normal vision from the University of Leuven Department of Psychology and Educational Sciences completed the experiment, either voluntarily or in exchange for course credit. The University of Leuven Department of Psychology and Educational Sciences Ethics Committee approved the experimental protocol and informed written consent was obtained from the subjects after the nature and possible consequences of the study were explained to them.

We selected a side view of a point-light walker from a different point-light stimulus set [45]. The height of each walker was 1.6 degrees of visual angle, whilst the width varied from 0.3 to 1.2 degrees, when viewed from 57 cm, the distance at which participants observed the stimuli. On half of the trials the walkers faced the direction in which they moved, whereas on the other half the walkers faced away from the direction they moved towards and therefore made an unnatural moon-walking motion across the screen.

Participants completed 6 practice trials and then 48 test trials. On each trial, participants were presented with 10 point-light walkers, 5 of which were briefly colored red, the rest of which were white. The walkers all appeared on one side of the screen for 533 ms, and began to walk towards a random location on the other side, following a smooth, although randomly determined trajectory. After another 533 ms the walkers initially colored red also turned white. All 10 point-light walkers then continued to move towards the other side of the screen for approximately 8.5 seconds (this time varied from trial to trial, with a standard deviation of 350 ms). The walkers moved at an average speed of 2.8 degrees per second, which was chosen such that the distance covered by the walker appeared natural with respect to the distance moved by the feet of the walker. When the walkers stopped at the other side of the screen a number was presented next to each walker, and the participant had to type in 5 numbers for the walkers that originally appeared in red. Participants were instructed to guess if they were unsure.

#### Results: Experiment 2

There was no hint of a difference between tracking accuracy between walkers and moon-walkers. Performance for the two conditions was essentially identical, with participants scoring on average 86.29% (SD = 6.23) for normal walkers and 86.63% (SD = 6.91) for moon-walkers. A paired samples t-test revealed that these values did not significantly differ (t(19) = 0.161, p = 0.874).

#### Discussion: Experiment 2

Experiment 2 did not reveal any effect of the congruency between the implied and actual direction of motion of point-light walkers on MOT. Thus this aspect of the naturalness of biological motion perception seemed to not influence object-based selection in MOT. Why inversion, but not moon-walking should influence MOT is not immediately apparent (although see Discussion). It appears that when presented in the canonical orientation, the set of dots that define biological motion can facilitate the grouping of those dots as an object of attention, but there was no special effect of the ecological validity of the motion *per se* in MOT.

### Introduction: Experiment 3

Inversion effects are perhaps most commonly associated with face stimuli [46], [47]. Given a general advantage across many tasks for upright faces and the advantage found for upright biological motion figures in Experiment 1, it seems logical that upright faces should also show an advantage in MOT. However, a recent study on the processing of identity in the tracking of faces showed that tracking was actually easier for inverted faces [48]. These authors suggested that when upright, a face is likely to be automatically processed in terms of its identity, which could interfere with its tracking (for other possible interpretations see Discussion). However, due to its focus on facial identity, this study did not explore tracking of identical targets. Here, we compared tracking performance for identical upright and inverted faces, as well as for upright and inverted houses, selected as a control stimulus that is not biological and is less sensitive to inversion.

#### Materials and Methods: Experiment 3

Twenty-eight adults (17 females, Mean age = 25.1, SD = 4.8) with normal or corrected-to-normal vision took part in this study. Eight were recruited from the student pool at the University of California, San Diego and 20 completed the experiment voluntarily at the University of Leuven. The University of Leuven Department of Psychology and Educational Sciences Ethics Committee and the UCSD Institutional Review Board approved the experimental protocol and informed written consent was obtained from the subjects after the nature and possible consequences of the study were explained to them.

One face and one house stimulus from a standard fMRI localizer stimulus set were used [49]. Each grayscale image contained 78×88 pixels and subtended 2.3 by 2.55 degrees of visual angle when viewed at 57 cm.

Stimuli were presented using Matlab (Mathworks, Natick, MA, USA) and the Psychophysics Toolbox [39], [40]. Participants completed 4 practice trials and then 48 test trials. On each trial, participants were presented with 9 images from one of the 4 stimulus types listed above. Participants completed an equal number of each trial type in a randomly determined order. At the start of each trial, the 4 targets were highlighted by a framing red line in one corner for 1143 ms (80 frames at 70 Hz). All 9 items then began to move, with the red line outlining the MOT targets for another 571 ms (40 frames). The red mark was then removed and all of the items continued moving randomly across the screen for 6571 ms (460 frames). The targets and distracters moved at an average speed of 2.8 degrees per second. At the end of the trial, a number was presented in the centre of each image, and the participant was instructed to press the numbers associated with the targets highlighted in red at the start of the trial.

#### Results: Experiment 3

**Figure 3:**
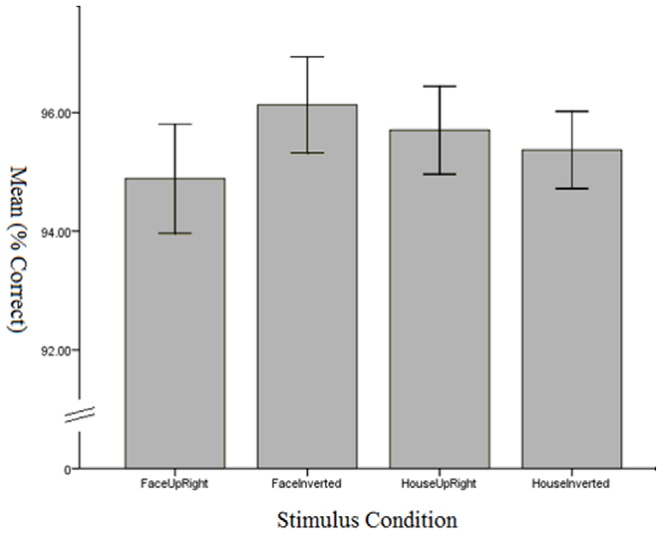
Accuracy for upright and inverted and face and house stimuli. Error bars are standard error. Inverted faces were tracked significantly more accurately compared with upright faces (see Results: Experiment 3).

Tracking accuracy for the 4 target types are shown in Figure 3. A paired samples t-test revealed that the tracking of inverted faces was more accurate than the tracking of upright faces (t(27) = 2.45, p = 0.021). There was no difference between upright and inverted houses (t = 0.364). A repeated measures ANOVA revealed no overall difference between tracking accuracy for faces and houses (F(1,27) = 0.002).

## Discussion

If one thinks of a football player mindful of the location of their teammates, or a mother trying to keep track of her children at the winter sales at Selfridges, it is evident that the tracking of other animate entities is both commonplace and important. Here, in 3 experiments, we explored the processing of biological stimuli in the Multiple Object Tracking (MOT) paradigm. (Please note that raw accuracies should not be compared between the experiments since several factors that can affect MOT performance such as the number of items to be tracked, tracking duration, field of view (e.g., see [32], [50]), and movement speed varied between the experiments as described in the Methods.)

Experiment 1 tested MOT for biological and non-biological point-light stimuli that were presented upright, inverted and scrambled. Overall, the letters were tracked slightly more accurately, likely due to a common fate advantage [51], and/or a processing cost disadvantage due to the additional internal motion signals inherent in the biological motion stimuli [42]. Scrambling significantly reduced tracking accuracy for both types of stimuli. In addition, there was a smaller, but reliable inversion effect specific for biological motion (replicated in a separate group of subjects in a pilot study). Inversion had previously been shown to affect processing of biological motion in such varied tasks as motion coherence [36] and audiovisual temporal judgments [37]. We now found that point-light biological motion figures are also more effectively tracked when presented in their canonical orientation.

There are some differences between inversion and scrambling that need to be considered in relation to MOT. For example, whereas scrambling affects generic grouping principles, inversion does not change the manner in which the dots can be grouped in terms of Gestalt principles as radically (e.g., proximity, good continuation). Inversion in turn changes the way the percept can be matched to previous exemplars of that specific stimulus type, which are overwhelmingly upright in this case [38]. Inversion also alters the gravitational information in the local motions, which has been argued to be a key cue to animacy [44].

Whilst these results do not support a strong specialization of MOT for biological motion, MOT does appear to be sensitive to the coherence of the tracked objects. Inverting and scrambling biological motion animations both disrupt the coherence of point-light stimuli, which in turn may make them less efficiently selected or maintained as objects of attention. Scrambling the point-lights of the letter stimuli should also have a similar effect as this creates an incoherent object. Inversion appeared to have little influence however on the tracking of point-light letters, most probably because strong grouping cues were maintained after inversion (e.g., the straight back and the semi-circle of the letter R, and the common fate of the moving dots, see Fig. 1), leading still to the percept of a coherent (albeit unfamiliar) object.

In this framework, the ease with which the visual system can group an object from individual elements will be intrinsically connected to the ease with which those objects can be tracked. Of course, tracking would not only be influenced by bottom-up grouping cues, but also by the allocation of attention. In this context, while our results show a difference in performance comparing coherent (here, upright) objects and less coherent (here, inverted and scrambled) objects, it is not possible to say whether this is due to an advantage for the coherent objects *per se*, or a disadvantage for the incoherent objects (e.g., because attentional resources are pulled away from the tracking task in order to keep the targets held together). Likely, both of these processes are intrinsic to MOT, as grouping can guide attention, and attention can facilitate grouping [5].

The effects of inversion and scrambling may be surprising with respect to some theories regarding the units of tracking in MOT. In particular, MOT has been argued to be underpinned by a limited set of proto-objects that are extracted in early vision in a manner that is entirely encapsulated from higher-level representations [21]. Instead, we found that MOT shows sensitivity to aspects of the stimuli being tracked that are very unlikely to be computed solely within early visual cortex [52]. Thus, either MOT has some direct access to the computations performed in higher areas, or that these higher level computations are fed back to and influence representations in levels at which MOT can select as targets. Higher-level grouping processes have been shown to influence early visual representations, even at the level of the primary visual cortex (e.g., [53], [54]).

Given the results of Experiment 1, we asked what other naturalistic aspects of biological stimuli MOT might be sensitive to. In our first experiment, our stimuli had repeated a star jump (jumping jack) action as they moved across the screen. In the real world of course, biological objects move in a manner that is consistent with the action they are performing. For example, if someone is facing leftward, they will in general be walking in that direction as well. In Experiment 2, we explored MOT with point-light walkers that walked naturally from one side of the screen to the other, and walkers that faced one direction, but moved in the other, i.e., moon-walking. The results showed no difference between participants' ability to track natural walkers and moon-walkers.

Thus, Experiment 2 revealed no advantage for the naturalness or ecological validity of walking biological motion stimuli. Comparing this to the inversion effect found in Experiment 1, one possible factor is frequency of exposure. Most people have never seen people walking on the ceiling [55], but have had a small amount of exposure to moon-walking (as well as to inverted letters). We think such experience is unlikely to override the effect however, except perhaps in the most dedicated Michael Jackson fans. Instead, we suggest that our results arise because MOT is sensitive to the ease with which the targets are selected and maintained as objects of attention, but not necessarily the ecological validity or naturalness of the stimulus motion *per se*.

In Experiment 3, we tested MOT using faces, another class of biological stimuli where inversion effects have been clearly demonstrated [46], [47], [58]. In contrast to the advantage found for upright biological motion figures in Experiment 1, Experiment 3, as well as a recent study [48], found inverted faces were more accurately tracked. Thus biological motion and face inversion effects were double dissociated in terms of their effects on MOT.

There are a few of possibilities that can help explain the effects of face inversion on MOT. Since MOT is not only a process of selecting targets, but also of inhibiting distracters (e.g., [56]), and since there is evidence that upright faces are harder to inhibit (e.g., [19]), the difference between inverted and upright faces in MOT may reflect the additional challenge in inhibiting the distracters (also faces). Thus the manner in which upright faces normally attract attention could in fact lead to a disadvantage in the context of MOT. A distinct, although related interpretation suggests upright faces automatically attract processing resources, which then detracts from the resources available for tracking [48]. It is however unclear why these explanations would not apply to upright biological motion figures, which show the opposite inversion effect.

As already discussed above, the most likely explanation for the inversion effect for biological motion pertains to the fact that inverted biological figures are likely to be perceived as a somewhat correlated swirl of dots, rather than a whole object. Thus inversion will decrease the extent to which the stimuli, comprising a collection of dots, are integrated into an object [51] and/or reduce the ability to ignore the otherwise distracting motion of its individual parts [42]. How is it possible to reconcile this increase in object coherence for biological motion with a decrease in tracking ability for faces? Functional magnetic resonance imaging (fMRI) studies have shown that face inversion leads to increased activity in extrastriate regions that respond preferentially to pictures of objects (houses) [57]. Specifically, an increase was found in the object-sensitive area LOC when viewing inverted faces [58], supporting the idea that faces may become more object-like when inverted, at least at some levels of processing. Thus, whilst the inversion of the face might reduce our ability to recognize and respond to it as a face, the same inversion might increase our ability to treat the face as an object of attention during tracking. This interpretation, although admittedly speculative, is consistent with a more general role for LOC in providing the units of selection in object-based attention tasks [14], [15].

### Discussion: Summary and Conclusion

We used the MOT paradigm with biological motion stimuli to explore a potential specialization for biological stimuli as objects of attentional selection. We assessed the tracking of point-light biological motion figures and point-light letters in upright, inverted and scrambled conditions. While we found effects of inversion and scrambling on MOT, these performance differences could be explained in terms of grouping factors rather than a specialization for biological stimuli. The finding that MOT shows some sensitivity to the higher-level status of the tracked items contrasts with theories of MOT that posit the indexing of proto-objects is achieved in early vision and is entirely encapsulated [21]. Next, we explored another naturalistic or ecologically valid feature of natural biological motion perception, by contrasting tracking performance for walkers who moved across the screen in a manner that was consistent with their internal motion profile, with artificial moon-walkers, whose direction of motion did not match the direction in which they faced. MOT was completely insensitive to this aspect of biological motion, suggesting the MOT is sensitive to the extent to which groups of dots can be segmented into one object, but not to the naturalness or ecological validity with which that object moves. Finally, we found that inverted faces were easier to track than upright faces, an effect that could reflect an inability to inhibit upright faces as distracters, an automatic allocation of resources to upright faces that detracts from tracking performance, or a shift to more generic object-based processing for inverted faces, the latter of which is easiest to reconcile with the opposite inversion effect found for biological motion.

Thus whilst MOT is sensitive to certain aspects of “biologicalness”, these sensitivities do not amount to a strong specialization for tracking biological, naturalistic, or ecologically valid stimuli. Instead, the MOT effects we observed can be framed in terms of the extent to which stimuli can be segmented, grouped and selected as targets of object-based attention. It appears that effectively grouped, coherent objects, but not necessarily biological objects, are tracked most successfully.
